# Public awareness and use of 997 emergency medical service phone number during the COVID-19 pandemic

**DOI:** 10.3389/fpubh.2022.937202

**Published:** 2022-10-03

**Authors:** Duaa Aljabri, Hissah Albinali

**Affiliations:** Health Information Management and Technology Department, College of Public Health, Imam Abdulrahman Bin Faisal University, Dammam, Saudi Arabia

**Keywords:** 997 phone number, awareness, COVID-19, emergency medical service (EMS), healthcare management, public health, Saudi Arabia

## Abstract

**Background:**

Emergency medical services (EMSs) are an important element of the healthcare system as it provides an opportunity to respond to critical medical conditions and save people's lives. In Saudi Arabia, EMS is offered *via* the EMS phone number “997” and mobile application “Asefny”.

**Methods:**

This was an observational cross-sectional survey study exploring public awareness and use of the EMS phone number during the COVID-19 pandemic in Saudi Arabia. A bivariate analysis was performed to investigate factors affecting awareness and use of the EMS phone number and to compare the EMS acceptance to transport and timelines of ambulance arrival between requests made *via* the “997” EMS phone number and the “Asefny” mobile application during the country's emergency lockdown.

**Results:**

A total of 805 participants were included in the analysis, where 66% reported awareness of the EMS phone number and 75% of them accurately identified the nature of the service provided by dialing the number. The men who participated, those with a bachelor's degree, with children, and with chronic conditions were more aware of the EMS phone number compared to the other participants. Of the total sample, 46.7% used EMS phone numbers at least one time (ever users). During the COVID-19 lockdown, the EMS accepted to transport 87% of the calls made by 997 phone number and 56.2% of the mobile application requests (*P* < 0.00). The ambulance arrived in ≤ 8 min in 53.6% of the 997 phone calls and 35.5% of the Asefny mobile requests (*P* < 0.00).

**Conclusions:**

Findings showed commendable levels of awareness and the use of EMS phone numbers. However, the results suggest room for improvement by developing promotional and educational campaigns inspired by the factors identified as influential on both awareness and use. Mobile applications in EMS are promising to improve prehospital emergency service accessibility, which needs to be further investigated to assess its impact on the public health informatics experience.

## Introduction

Emergency medical services (EMSs) are an important element of the healthcare system as it provides an opportunity to respond to critical medical conditions and save people's lives. Those conditions need rapid assessment and timely transportation to the nearest health facility to enhance survival and prevent disability (1).

Many countries in the world are working on improving EMS using phone services and mobile applications. For example, India primarily uses two EMS models to support service delivery; the 108 emergency service works as a response system for accident and trauma victims by providing critical care (2), and the 102 ambulance service offers basic patient transport to referral and home facilities (3). In the United States, EMS has steadily grown and reached a level where the federal government is working to ensure that all adults are aware of the services they can receive (4). Many Americans use mobile health applications rather than the phone services because they allow them to access their medical history, consult their doctors, receive prescriptions, and recommendations for nearby facilities in case of emergencies. In Saudi Arabia, the Saudi Red Crescent Authority (SRCA) is the national entity responsible to operate the EMS *via* a dedicated call number 997 and “Asefny”, i.e., “*Save me”* mobile application. The mobile application service was introduced in 2017 as part of the Saudi National Transformation Program and the Saudi Vision 2030 plan and provided *via* two-way communication between the SRCA dispatch center and the reporter using the short message service (5). This communication aids the dispatcher in triage and requests medical history that support decision-making for medical transport. The application also includes features to precisely locate the reporter, which saves time for the emergency team. It also provides locations of the closest health facilities to the user and other emergency numbers and the ability to build a profile of the user's medical history.

Markedly, the primary role of emergency services is to address public care needs within a short time possible. The International Standards Organization has adopted response time as a crucial indicator of the level of emergency services being delivered in local, national, and international settings (3). Many nations globally, including Saudi Arabia, use the 8 min interval between call receipt and arrival at the scene to determine the emergency medical services' effectiveness (6). However, while most people know the availability of EMS, only a handful understands this care's innate attributes (7). In addition, as the prehospital associated death rates have increased over recent years, that has raised several concerns regarding the awareness and utilization of ambulance services (8).

There is a gap in the literature in terms of examining the public awareness of the EMS phone number in Saudi Arabia. Only two studies were conducted to examine awareness; one in the western region (9), where 33% were unaware of the free-of-charge EMS number 997, and another recent study in Riyadh (7), where 73.2% claimed to know the EMS phone number and 38.5% recalled the number correctly. As Saudi Arabia is experiencing a series of pandemics in the past few decades such as MERS, EBOLA, and COVID-19, the demand for EMS has significantly increased (10). Thus, it is highly important that people's awareness of emergency numbers must be assessed at regular intervals. Furthermore, previous studies (8, 11–13) suggest that the mobile health applications can significantly improve emergency medical services, which can facilitate improved healthcare service accessibility. The previous studies focused mostly on the programs' content, nature, and formulation, thereby ignoring the community's awareness of the ambulance number and the proper use of the ambulance service in emergency cases. That being said, the literature gaps between the initiative formulation, people's awareness about it, and their use still exist. Therefore, the purpose of this study is to evaluate the public awareness and the use of the EMS phone number and to identify the factors affecting awareness and use. In addition, the study compares EMS response and timelines between requests made *via* the 997-phone number and “Asefny” mobile application during the COVID-19 lockdown in Saudi Arabia.

## Methods

The study is an observational cross-sectional study that targeted all people living in Saudi Arabia. A bilingual (Arabic and English) survey was developed and validated to measure the public awareness and use of the EMS phone number and “Asefny” mobile application. In the validation stage, the survey was sent to four academic experts for content validity. It was then pilot-tested by nine randomly selected individuals from the public to ensure clarity. An online survey link was created for the survey using Google surveys. Due to the COVID-19 pandemic, physical interaction with the participants would have posed considerable health risks. Thus, social media platforms such as WhatsApp and Twitter were used in the dissemination process. The survey had two sections: the questions in the first section were marked for one point, asking about participants' demographics, such as gender, age, nationality, geographic location, and education status. The questions in the second section collected information about participants' awareness and use of 997 EMS phone number, which were visible one by one with no chance to move between questions. This logic was built in the electronic survey to limit the introduction of bias in the results. Awareness was assessed by asking two questions: “Are you aware of the EMS phone number?” (Yes/No). Only if the respondent answers with yes does the second question appear, “Which of the following do you think 997 service provide?”, to assess true awareness (where responding to medical and trauma emergencies was the correct answer). Incorrect responses to the second question were considered false awareness about the EMS number.

The use was assessed by answering positively to “Have you ever called EMS for an ambulance?” (Yes/No). The survey was distributed in 2 months (October and November 2020), using the snowball sampling technique, where participants were requested to forward the survey link to their colleagues, friends, and families in order to increase the sample size. The total responses received were 936, out of which 131 were incomplete. After excluding incomplete responses, the valid responses for analysis were 805 responses.

Data analysis was carried out using the IBM^®^ SPSS^®^ software platform. The data analysis mainly involved the use of descriptive techniques such as percentages and frequencies. A bivariate analysis using the chi-square test was used to compare percentages and identify factors that may influence awareness and use. A *p*-value of < 0.05 was considered significant.

## Results

The total number of respondents of the survey was 805. Table 1 presents the demographic characteristics of the sample. Most participants in the sample speak Arabic as their primary language (94.9%, *n* = 764). The sample was balanced from a gender perspective, with men and women participants contributing to 52.5% (*n* = 423) and 47.5% (*n* = 382) of the sample, respectively. A review of the age distribution in the sample shows that it mirrors the national trends, with persons aged over 60 y only representing 2.1% (*n* = 17) of the sample. The age groups 20–29 (33.2%, *n* = 267) and 30–39 (37.6%, *n* = 303) had the highest representation in the sample. All of the responses were from Saudi citizens (92.2%, *n* = 748), while 7.1% (*n* = 57) were residents. Responses from all five geographic regions were included in the sample. However, the Eastern region appears to be overrepresented, being 46% (*n* = 370) of all the participants. Only 10.9% (*n* = 88) of the respondents reported having at least one chronic disease.

**Table 1:** Study sample characteristics (*N* = 805).

**Variable**	**N**	**%**
**Gender**		
Female	382	47.5
Male	423	52.5
**Age**		
15–19	26	3.2
20–29	267	33.2
30–39	303	37.6
40–49	142	17.6
50–59	50	6.2
60+	17	2.1
**Nationality**		
Residents (Non-Saudi)	57	7.1
Citizens (Saudi)	748	92.9
**Geographic location**		
Middle region	172	21.4
Eastern region	370	46.0
Northern region	88	10.9
Southern region	92	11.4
Western region	83	10.3
**Education**		
High school	178	22.1
Diploma	113	14.0
Bachelor degree	442	54.9
Graduate studies	72	8.9
**Having children** (Yes)	446	55.4
**Having chronic disease** (Yes)	88	10.9

The results (Table 2) revealed that 66% (*n* = 531) of the participants were aware of the 997 EMS phone number. Of those, 24.6% (*n* = 131) had false awareness, as they were unaware of the nature of the service provided by 997. In addition, 26.8% (*n* = 216) of the participants reported that they had called the wrong emergency number for help. About half of the participants (46.7%, *n* = 367) requested emergency services at least one time (ever-users). During the COVID-19 lockdown, 60.9% (*n* = 229) of ever-users have requested an ambulance; 41% (*n* = 94) by dialing 997, and 59% (*n* = 135) by requesting services from Asefny mobile application.

**Table 2:** Public awareness and use of EMS phone number (*N* = 805).

**Questions**	**N**	**%**
**Awareness 1: Are you aware of the EMS phone number?**		
No	274	34.0
Yes	531	66.0
**Awareness 2: Which of the following do you think the 997 phone number provides? (*****N** **=*** **531)**		
Medical appointments	23	4.3
Respond to medical & trauma emergency (correct answer)	400	75.3
Medical consultations	52	9.7
Road assistance service	30	5.6
I do not know	26	4.8
**Have you ever had an emergency and called the wrong number for help?**		
No	589	73.2
Yes	216	26.8
**Use: Have you ever called EMS for an ambulance?**		
No	429	53.3
Yes	376	46.7
***Have you requested EMS for an ambulance during COVID-19 lockdown? (N** **=** **376)***		
Yes	229	60.9
***Mode of request (N** **=** **229)***		
*Calling 997*	94	41.0
*Asefny mobile application*	135	59.0

Table 3 shows separate bivariate analyses using the chi-squared test, between the awareness and use of the EMS phone number, separately, and the study of independent variables. The awareness was significantly associated with gender, geographic location, nationality, education, having children, and having a chronic illness (*p* < 0.05). The use was significantly associated with gender, age, geographic location, and having children (*p* < 0.001).

**Table 3:** Bivariate analysis of awareness and use of 997 EMS phone number.

	**Aware^a^** ***N =* 400 (%)**	**Not aware** ***N =* 274 (%)**	***P***	**Users** ***N =* 376 (%)**	**Non-users ** ***N =* 429 (%)**	***P***
**Gender**			**< 0.001**			**< 0.001**
Female	164 (41.0%)	195 (52.7%)		146 (38.8%)	236 (55.0%)	
Male	236 (59.0%)	175 (47.3%)		230 (61.2%)	193 (45.0%)	
**Age**			0.17			**< 0.001**
15–19	12 (2.8%)	14 (3.8%)		1 (0.3%)	25 (5.8%)	
20–29	144 (36.0%)	106 (28.6%)		102 (27.1%)	165 (38.5%)	
30–39	140 (35.0%)	152 (41.1%)		155 (41.2%)	148 (34.5%)	
40–49	69 (17.3%)	69 (18.6%)		77 (20.5%)	65 (15.2%)	
50–59	28 (7.0%)	21 (5.7%)		36 (9.6%)	14 (3.3%)	
60+	7 (1.8%)	8 (2.2%)		5 (1.3%)	12 (2.8%)	
**Geographic location**			**< 0.001**			**< 0.001**
Middle region	91 (22.8%)	69 (18.6%)		69 (18.4%)	103 (24.0%)	
Eastern region	217 (54.3%)	141 (38.1%)		157 (41.8%)	213 (49.7%)	
Northern region	29 (7.3%)	53 (14.3%)		53 (14.1%)	35 (8.2%)	
Southern region	34 (8.5%)	57 (15.4%)		55 (14.6%)	37 (8.6%)	
Western region	29 (7.3%)	50 (13.5)		42 (11.2%)	41 (9.6%)	
**Nationality**			**0.01**			0.31
Residents (Non-Saudi)	19 (4.8%)	35 (9.5%)		23 (6.1%)	34 (7.9%)	
Citizens (Saudi)	381 (95.3%)	335 (90.5%)		353 (93.9%)	395 (92.1%)	
**Education**			**< 0.001**			0.22
High school	68 (17.0%)	105 (28.4%)		81 (21.5%)	97 (22.6%)	
Diploma	45 (11.3%)	64 (17.3%)		63 (16.8%)	50 (11.7%)	
Bachelor degree	239 (59.8%)	179 (48.4%)		200 (53.2%)	242 (56.4%)	
Graduate studies	48 (12.0%)	22 (5.9%)		32 (8.5%)	40 (9.3%)	
**Having children (Yes)**	238 (59.5%)	191 (51.6%)	**0.04**	231 (61.4%)	215 (50.1%)	**< 0.001**
**Having chronic disease (Yes)**	61 (15.3%)	24 (6.5%)	**< 0.001**	46 (12.2%)	42 (9.8%)	0.26

a Awareness is based on knowing the service provided by 997 accurately. Participants unable to recall the correct service (i.e. respond to medical and trauma emergency) were excluded from this group (*n* = 131).

The bold values indicate the significant association.

Table 4 presents a comparison of the use of 997 and the Asefny mobile application during the COVID-19 lockdown. Differences were found, where the type of emergency, responding with an ambulance, and response time ( ≤ 8 min) were significantly different between the two EMS modes of request.

**Table 4:** Comparison of the purpose and response time of EMS service requested by phone number vs. mobile application during the COVID-19 lockdown.

	**Total** ** (*N =* 229) *n* (%)**	**997 phone number** ** (*N =* 94) *n* (%)**	**Asefny mobile application (*N =* 135) *n* (%)**	***P***
**Type of emergency**				**< 0.001**
Medical illness	79 (34.4)	21 (22.3)	58 (42.9)	
Violence	26 (11.3)	17 (18.0)	9 (6.6)	
Trauma	38 (16.5)	27 (28.7)	11 (8.1)	
Burn	13 (5.6)	10 (10.6)	3 (2.2)	
Suicide attempt	2 (0.8)	2 (2.1)	0 (0)	
Unconsciousness	15 (6.5)	12 (12.7)	3 (2.2)	
Request permit to travel during curfew	56 (24.4)	5 (5.6)	51 (37.7)	
**Responded with an ambulance (Yes)**	158 (68.9)	82 (87.2)	76 (56.2)	**< 0.001**
Ambulance arrived in ≤ 8 min (Yes)	71 (31.0)	44 (53.6)	27 (35.5)	**< 0.001**

The bold values indicate the significant association.

## Discussion

The study aimed to assess the awareness and use of the 997-EMS number during the COVID-19 pandemic in Saudi Arabia. The study also investigated factors that may affect the awareness and use of the 997-EMS number and compared the type of emergencies and response to requests made by phone vs. the mobile application during the lockdown. The findings revealed that 66% reported awareness about the 997, while only 24% were not able to recall the service provided by 997 number correctly. Regardless of false awareness, public awareness in this study is considered the highest among awareness trends studied previously in the country; 33% in 2015 and 38.5% in 2021 (2, 3). In addition, the percentage of 997 ever-users was 46.7%, where 61% of them requested an ambulance during the lockdown. This rate is considerably higher than those reported in the United Kingdom, where the NHS 911 service utilization rate was 9% (14).

Such an increase in the rate of awareness and use is subject to the COVID-19 pandemic and lockdown. The first case of COVID-19 in Saudi Arabia was confirmed on 2 March 2020. On 23 March 2020, an order was issued by the government that restricted movement from 1900 hrs to 0600 hrs. These restrictions lasted until 28 June 2020, when the government began implementing its return to normal policy plan.

It is important to note that almost 27% reported calling the wrong emergency number for help. As several three-digit numbers for public service agencies in Saudi Arabia exist; for example, medical consultations and appointments is 937, the police is 999, and the civil defense is 998; this may create confusion among the people, especially among those who had false awareness in this study.

The study showed that awareness could be influenced by gender, geographic location, nationality, education, having children, and having a chronic illness. Having chronic illness and children is likely to lead to increased interest in and awareness of health information, including emergency services (15). Education can affect awareness of the emergency number *via* health literacy and information-seeking behavior (16). Individuals in urban areas are usually more aware of the service compared to those in remote regions due to the high accessibility to healthcare services (17). The Eastern region, which is predominantly urban, had the highest rates of both awareness and the use of the emergency service number, which is in line with this expectation. Gender differences in health literacy within Saudi Arabia could explain the role of gender in both awareness and its use (18).

The study shows that there are differences in the modes of requesting EMS: dialing 997 or sending a message *via* Asefny mobile application. First, the use of Asefny mobile application was higher than the use of the 997 number. Second, there was also a higher preference to use the Asefny application over calling 997 during the lockdown and curfew. There was also a noticeable difference in the type of emergency requested in the two modes: Asefny was preferred in cases of medical illness and when seeking a permit. A permit from EMS can be used to transport the patient to the nearest hospital by the service requester, whereas calls to 997 were mainly observed in case of emergency situations such as violence, trauma, burn, and suicide attempts.

There were significant differences in both the response to the calls or requests and the timeliness of the responses. The ambulance service responded more to the 997-emergency service than they did for requests made *via* the mobile application because the requests over the phone may be more critical to analyze as the caller may be under stress. Second, the ambulances were more likely to respond in less than 8 min for those requests made *via* the 997-emergency line when compared to the requests made *via* the mobile application. However, in a similar study comparing responses through 997 and Asefny, no differences in the response times were identified (10). The differences in both the response to the calls or requests and the timeliness of the responses may be due to the extensive infrastructure behind the 997-emergency service. The interval between scene arrival and call receipt is the commonly accepted and used parameter in Saudi Arabia to discern the quality of emergency services being offered (8, 10). Many countries globally deploy necessary efforts to achieve the response time of 8 min, set by the International Standards Organization (3). The findings suggest that the 997-emergency service is outperforming the mobile application in terms of the proportion of responses to requests and the timeliness of the responses.

### Strengths and limitations

The study used a large sample size that enhanced its reliability and replication among different groups of participants. The 805 participants selected using the recruitment procedure and ethical consideration would eliminate bias from the researcher's end and encourage the provision of accurate data by participants. The use of an online survey improved the confidence of the participants who would feel intimidated to respond in face-to-face interviews. Despite these strengths, the study has some limitations. First, the most significant limitation is recall bias since we did not collect data during the pandemic lockdown period and have asked for data retrospectively. Second, half of the participants in the study were from the eastern region. Thus, there is the risk that the views of participants from this region can pass for the views of Saudi residents. Third, the cross-sectional survey design of this study does not allow for cause-and-effect inferences. From the study, it cannot be concluded that having a chronic illness causes one to be more aware of the 997-emergency service. Thus, future scientific studies should seek to determine whether the observed association between the use or awareness rates and gender, education, geographical region, having a child, and having a chronic condition constitute a cause-and-effect relationship. Furthermore, future studies should endeavor to use geographically balanced samples by employing stratified sampling. Also, further investigations on the barriers to using the mobile application are recommended.

### Study implications

This study has both theoretical and practical implications, which emphasize the importance of findings in this study. First, this study contributes to the current health emergency literature related to public awareness of health emergency numbers, the use of health emergency applications, and the response times from both approaches. Second, the changes observed in this study with respect to awareness levels compared to the previous studies can enable practitioners and decision-makers to taking appropriate decisions. For instance, assessing the issues for higher response times for requests made through Asefny highlights the need to re-engineer and redesign the existing processes to improve the response times. In addition, the study has implications for policymakers to promote public awareness and usage rates to maximize the value of this service. As most public agencies in the country have a free-of-charge 3-digit number, the EMS number can be easily confused with other services and cause a delay in reaching the needed service (19). The media can contribute to awareness by education campaigns or short films and scenarios that are well-perceived by the community of different age, educational backgrounds, geographic regions, and medical illnesses. For instance, the media can endeavor to popularize the emergency service in predominantly rural regions, among persons with chronic conditions, and with lower levels of education. The findings of this study can support targeting the groups that have displayed lower levels of EMS awareness or use by eliminating barriers to the service while enhancing the awareness and usage statistics.

## Conclusion

The main aim of the study was to investigate public awareness and use of the EMS phone number in Saudi Arabia and identify factors affecting awareness and use during the pandemic. We found that 66% reported awareness about the 997 phone number and 46.7% were ever-users. Being male and having children were significant factors associated with both awareness and use of the 997 phone number. During the COVID-19 pandemic, we observed that Asefny emergency requests were more common than those made *via* 997, suggesting the usefulness of mobile applications as a supplementary EMS service. Yet, 87% of the calls made by 997 were accepted for medical transport while only 56.2% of the Asefny requests were accepted. More medical-related emergencies were made *via* 997 while the Asefny was more commonly used for travel permits. To our knowledge, this is the only study to date that explored the acceptance of medical transport and timeliness of ambulance arrival between requests made through the EMS phone number vs. mobile application during the pandemic lockdown at a national level. The study provides insights for policymakers in planning strategies for the effective use of mobile applications in emergencies to support prehospital care.

## Data availability statement

The raw data supporting the conclusions of this article will be made available by the authors, without undue reservation.

## Ethics statement

The ethical approval was obtained from the Institutional Review Board of Imam Abdulrahman Bin Faisal University, Dammam, Saudi Arabia (IRB-PGS-2021-03-049). Participants provided their written informed consent to participate in this study.

## Author contributions

All authors listed have made a substantial, direct, and intellectual contribution to the work and approved it for publication.

## Conflict of interest

The authors declare that the research was conducted in the absence of any commercial or financial relationships that could be construed as a potential conflict of interest.

## Publisher's note

All claims expressed in this article are solely those of the authors and do not necessarily represent those of their affiliated organizations, or those of the publisher, the editors and the reviewers. Any product that may be evaluated in this article, or claim that may be made by its manufacturer, is not guaranteed or endorsed by the publisher.
